# Implication of the cooking oil-peroxidation product “hydroxynonenal” for Alzheimer’s disease

**DOI:** 10.3389/fnagi.2023.1211141

**Published:** 2023-08-25

**Authors:** Tetsumori Yamashima, Takuya Seike, Daria Mochly-Rosen, Che-Hong Chen, Mitsuru Kikuchi, Eishiro Mizukoshi

**Affiliations:** ^1^Department of Psychiatry and Behavioral Science, Kanazawa University Graduate School of Medical Sciences, Kanazawa, Japan; ^2^Department of Gastroenterology, Kanazawa University Graduate School of Medical Sciences, Kanazawa, Japan; ^3^Department of Chemical and Systems Biology, Stanford University School of Medicine, Stanford, CA, United States

**Keywords:** amyloid β, *calpain–cathepsin hypothesis*, Hsp70.1, lysosome, neuronal death, PUFA

## Abstract

Aldehyde dehydrogenase 2 (ALDH2) is a mitochondrial enzyme that reduces cell injuries via detoxification of lipid-peroxidation product, 4-hydroxy-2-nonenal (hydroxynonenal). It is generated exogenously via deep-frying of linoleic acid-rich cooking oils and/or endogenously via oxidation of fatty acids involved in biomembranes. Although its toxicity for human health is widely accepted, the underlying mechanism long remained unknown. In 1998, Yamashima et al. have formulated the “*calpain–cathepsin hypothesis*” as a molecular mechanism of ischemic neuronal death. Subsequently, they found that calpain cleaves Hsp70.1 which became vulnerable after the hydroxynonenal-induced carbonylation at the key site Arg469. Since it is the pivotal aberration that induces lysosomal membrane rupture, they suggested that neuronal death in Alzheimer’s disease similarly occurs by chronic ischemia via the calpain–cathepsin cascade triggered by hydroxynonenal. For nearly three decades, amyloid β (Aβ) peptide was thought to be a root substance of Alzheimer’s disease. However, because of both the insignificant correlations between Aβ depositions and occurrence of neuronal death or dementia, and the negative results of anti-Aβ medicines tested so far in the patients with Alzheimer’s disease, the strength of the “*amyloid cascade hypothesis*” has been weakened. Recent works have suggested that hydroxynonenal is a mediator of programmed cell death not only in the brain, but also in the liver, pancreas, heart, etc. Increment of hydroxynonenal was considered an early event in the development of Alzheimer’s disease. This review aims at suggesting ways out of the tunnel, focusing on the implication of hydroxynonenal in this disease. Herein, the mechanism of Alzheimer neuronal death is discussed by focusing on Hsp70.1 with a dual function as chaperone protein and lysosomal stabilizer. We suggest that Aβ is not a culprit of Alzheimer’s disease, but merely a byproduct of autophagy/lysosomal failure resulting from hydroxynonenal-induced Hsp70.1 disorder. Enhancing ALDH2 activity to detoxify hydroxynonenal emerges as a promising strategy for preventing and treating Alzheimer’s disease.

## Background

**FIGURE 1:**
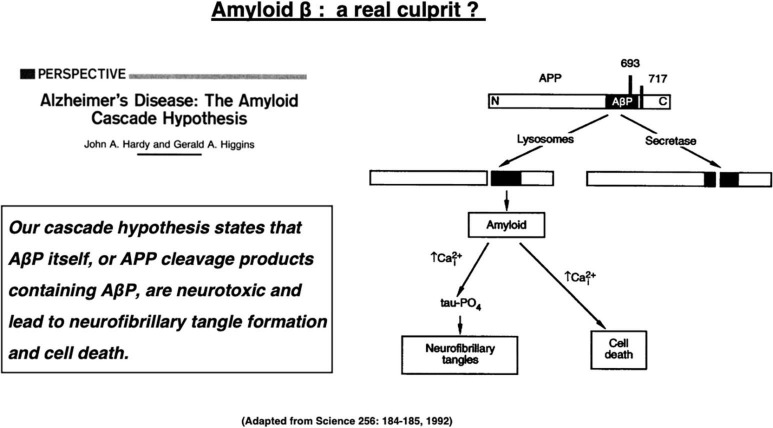
The “*amyloid cascade hypothesis*” was reported in 1992 by Hardy and Higgins as a perspective review in *Science*. Adapted from Hardy and Higgins (1992).

Alzheimer’s disease is the most popular dementia that affects more than 50 million people globally in 2018 (Collaborators GBDD, 2019). The number of patients with Alzheimer’s disease will increase to 152 million by 2050 (Patterson, 2018). Alzheimer’s disease is a chronic neurodegenerative disorder that is clinically characterized by disturbances of memory, cognition, and behavior. This disease is caused by widespread neuronal death in the hippocampus and associated neocortical regions, but the molecular mechanism underlying neuronal death has been unknown for more than a century. The Alzheimer brain bears two representative hallmarks: amyloid β (Aβ) plaques forming outside cells and neurofibrillary tangles (NFTs) forming inside neurons. Depositions of both proteins were first described as senile plaques by Dr. Alois Alzheimer more than a century ago. However, it was not until 1984 that Glenner and Wong demonstrated that Aβ is derived from the cell membrane protein called amyloid precursor protein (APP) (Glenner and Wong, 1984). In 1991, Hardy’s group has uncovered the first mutation of the *APP* gene which, they expected, would be responsible for the occurrence of the familiar Alzheimer’s disease (Hardy and Allsop, 1991; van Duijn et al., 1991). This study triggered the “*amyloid cascade hypothesis*” (called here “amyloid hypothesis”) in 1992 as an etiology of the familiar Alzheimer’s disease (Figure 1; Hardy and Higgins, 1992). Since the familiar form was almost indistinguishable from the sporadic form, the amyloid hypothesis was applied to both forms of Alzheimer’s disease.

For three decades since then, Aβ peptides were thought to be a root substance of neuronal death. Much of our knowledge on Aβ pathophysiology actually derives from the basic research using the transgenic Alzheimer model mice that generate abundant Aβ in the brain. However, its relevance to human Alzheimer’s disease should be reexamined now. We should keep in mind that the biomolecular role of Aβ in humans has not been elucidated in detail, even if using the transgenic mice model. In particular, in the patients with Alzheimer’s disease the following issues still remain unelucidated: (1) Why abundant Aβ depositions are present in the individuals with normal cognition? (2) Why some individuals diagnosed as Alzheimer’s disease exhibit not Aβ pathology but non-Aβ pathologies? (3) Why the spatial distribution, progression and extent of amyloid plaque depositions do not always correlate with cognitive decline? and (4) Why the number and location of Tau tangles are linked more closely to the cognitive disorders? Because of the less significant correlations between the extent of Aβ depositions and either degree of synapse pathology, neuronal loss or dementia, and also of all the negative results of the anti-Aβ clinical trials so far in the patients with Alzheimer’s disease, it seems prudent to conclude that the amyloid hypothesis has become unreliable (Pimplikar et al., 2010; Robakis, 2010; Makin, 2018; Yamashima, 2021).

The amyloid hypothesis has dominated research on Alzheimer’s disease for decades. Although most researchers propose that Aβ accelerates the formation of Tau tangles, which makes more damage to neurons than Aβ, drugs attacking Tau also failed. Even though the causal link between Aβ and neurodegeneration has not been established, and many possible Aβ-independent disease pathways could be more critical in the pathology, most researchers still place Aβ as a central to Alzheimer’s disease. We suggest that it is time to explore other molecular mechanisms that contribute to this pathology. Since the brain is rich in polyunsaturated fatty acids (PUFA) in the lipid bilayer and contains high concentration of oxygen, and since oxidative stress-induced lipid peroxidation results in accumulation of toxic aldehyde products, many studies now associate accumulation of these aldehydes with age-related neurodegeneration (Humphries et al., 1998; Uchida, 2003; Lashin et al., 2006; Yamashima and Oikawa, 2009; Yamashima, 2013, 2016, 2021; Ayala et al., 2014; Bekyarova et al., 2019; Gianazza et al., 2019; Gadhave et al., 2020).

**FIGURE 2:**
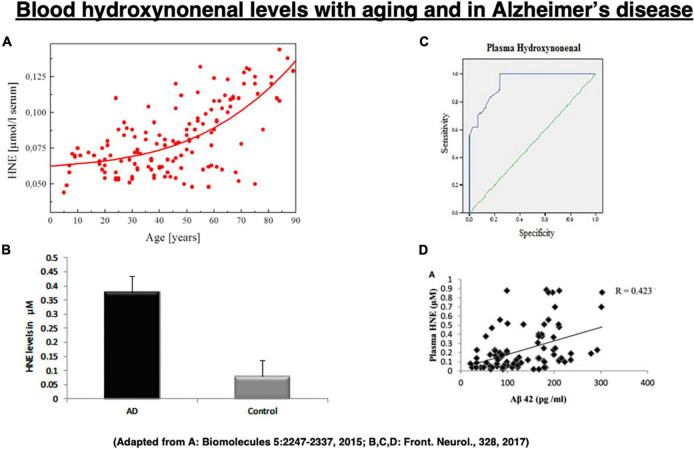
Change of the blood hydroxynonenal (HNE) levels with aging and in Alzheimer’s disease. HNE levels gradually increase after 40 years old in the healthy individuals, due to the age-dependent decrease of catalyzing enzymes **(A)**. HNE levels are much higher in the patients with Alzheimer’s disease (AD), compared to the healthy subjects (Control) **(B)**. Receiver operating characteristic curve analysis indicates that HNE is a better indicator of lipid peroxidation in Alzheimer’s disease **(C)**. Correlation analysis exhibits a positive correlation for HNE with Aβ_42_ **(D)**. **(A)** Adapted from Schaur et al. (2015); **(B–D)** adapted from Rani et al. (2017).

Among the lipid peroxidation products, 4-hydroxy-2-nonenal (hydroxy: -OH, -2-: two carbon double bonds, none: 9 carbon atoms, nal: aldehyde) (called “hydroxynonenal” here) represents one of the most bioactive lipid alkenals that impede cell health via forming covalent adducts with nucleophilic functional groups in proteins, nucleic acids, and membrane lipids. Hydroxynonenal levels in the serum gradually increase in the healthy individuals after 40 years old (Figure 2A), and are higher in Alzheimer’s disease patients relative to healthy individuals (Figure 2B). Further, its adducts are concentrated in Aβ plaques with mutual correlation (Figure 2D; Siegel et al., 2007; Butterfield et al., 2010). Here, we focus on the implications of oxidative stress and lipid-peroxidation product “hydroxynonenal” (Figure 2C) in the development of Alzheimer neuronal death, because recent studies showed its cell toxicity in various organs including brain (Yamashima, 2023), pancreas (Boontem and Yamashima, 2021), liver (Seike et al., 2022), and heart (Sun et al., 2014).

## “Amyloid hypothesis” was merely “perspective” at 1992

Since the discovery of Aβ in 1984 (Glenner and Wong, 1984), research on Alzheimer’s disease had focused on the detrimental role of this small peptide (Ricciarelli and Fedele, 2017), although its beneficial roles were repeatedly reported thereafter. Already in 1990, for example, Yankner et al. (1990) showed that Aβ is neurotrophic to immature hippocampal neurons. In 1995, Aβ_40_ was reported to enhance hippocampal long-term potentiation (LTP) in the dentate gyrus of rats (Wu et al., 1995). Tamaoka et al. (1997) demonstrated that Aβ is physiologically produced by neurons, although the cerebrospinal fluid (CSF) levels of Aβ_40_ are significantly lower in the normal subjects than Alzheimer patients. In 2008, it was found that even picomolar levels of Aβ_42_ can enhance hippocampal LTP in the normal mice, which was associated with the improvement of scores, using the Morris water maze and the fear conditioning tasks (Puzzo et al., 2008). Importantly, Aβ is physiologically produced in the brain, as indicated by its presence in the normal mice (Cirrito et al., 2003) and in the CSF of healthy individuals (Mehta and Pirttila, 2005; Giedraitis et al., 2007). These observations led to such an idea that Aβ may play a functional and positive role in the physiology of the brain (Pearson and Peers, 2006).

“*Our (amyloid) cascade hypothesis states that A*β*P (amyloid* β *protein) itself, or APP cleavage products containing A*β*P, are neurotoxic and lead to neurofibrillary tangle formation and cell death*,” described by Hardy and Higgins (1992) (Figure 1).

These words represented the dawn of the amyloid hypothesis that dates back in 1992 when they first posited Aβ peptides as a culprit of Alzheimer’s disease (Hardy and Higgins, 1992). Their hypothesis was based on the two *in vitro* evidence available at that time, i.e., (1) Aβ-containing COOH-terminal fragment is toxic to cultured neurons (Neve et al., 1990), and (2) Aβ alone exerts toxic effects on neurons, possibly through the serpin receptor (Roher et al., 1988; Kowall et al., 1991). “*Although it is not clear exactly how A*β*P causes neuronal loss and tangle formation, the peptide is known to disrupt calcium homeostasis and increase intraneuronal calcium concentrations* (Figure 1),” described by Hardy and Higgins (1992). The amyloid hypothesis had been strongly supported by four conceptually important observations (Fedele et al., 2015). (1) Mutations in three genes that are involved in the generation of Aβ (*APP, PS_1_*, and *PS*_2_) cause familial Alzheimer’s disease. (2) *APP* gene is localized at chromosome 21, and some patients with Down syndrome due to its trisomy develop Alzheimer’s disease early in life. (3) Aβ exerts cytotoxic effects on the *in vitro* cell systems. (4) Aβ accumulation in the senile plaques is an obligate feature for the diagnosis of Alzheimer’s disease.

Nowadays, however, available evidence (Aizenstein et al., 2008; Yamashima, 2013, 2016, 2021; Yamashima et al., 2023) opposes the amyloid hypothesis, because Aβ accumulation and depositions do not always correlate with neuronal death and cognitive impairments (Katzman et al., 1988; Delaère et al., 1990; Dickson et al., 1992). For instance, PET scan detecting Aβ disclosed that (1) many individuals have significant amyloid plaque burden without showing memory or cognitive impairments. (2) No correlations were observed between brain/CSF Aβ_42_ with neuropsychological test performance (Fagan et al., 2009; Klunk et al., 2009; Villemagne et al., 2011; Ricciarelli and Fedele, 2017). (3) Although Aβ oligomers are known to damage neurons *in vitro* (Lambert et al., 1998; Kim et al., 2003; De Felice et al., 2007; Ono et al., 2009; Ahmed et al., 2021), neuronal cell death is virtually absent in *APP* or *APP/PS_1_* transgenic mice modeling human Alzheimer’s disease. (4) Endogenous Aβ peptides, regardless of the assembly state, do not trigger neurodegeneration *in vivo* (Aizenstein et al., 2008). In addition, (5) Aβ and Tau proteins act in a synergistic fashion to cause neuronal death (Ittner and Götz, 2011), but distribution of NFTs and Aβ plaques do not occur simultaneously in the brain of Alzheimer patients, and tangles seem to precede plaque formation (Braak and Braak, 1991; Price et al., 1991; Schönheit et al., 2004). Surprisingly, however, a majority of the previous studies had continued focusing on the neurotoxic concentrations of Aβ, which are much higher than the physiological levels.

In summary, the demonstration of neuroprotective functions of Aβ, together with the failures of anti-Aβ clinical trials to show benefit, go against the amyloid hypothesis.

## Diagnostic criteria of Alzheimer’s disease require reassessment

The diagnostic criteria of Alzheimer’s disease and guidelines for preclinical research are still based on the amyloid hypothesis of disease etiology and associated assumptions (Albert et al., 2011; Sperling et al., 2011). The first assumption is that Aβ and Tau pathology are specific markers of Alzheimer’s disease. Second, that Alzheimer’s disease is a homogenous disorder in which individuals with familial or sporadic Alzheimer’s disease have the same character. The latter assumption has been widely accepted, because familiar (early-onset: ∼5% of cases with Alzheimer’s disease) and sporadic (late-onset: ∼95% of the cases) Alzheimer’s diseases are similar. The impact of discovering mutations of *APP, PS_1_*, and *PS*_2_ genes was profound, but the relevance of data from the observational and therapeutic studies with autosomal dominant mutation carriers to sporadic Alzheimer’s disease has gradually become less tenable (Morris et al., 2018). Further, the literature indicates that neither amyloid plaques nor NFT depositions are peculiar to Alzheimer’s disease (Jack et al., 2018). Dr. Alois Alzheimer and his contemporaries already noticed similarities in the clinical and pathological characteristics of syphilitic dementia and Alzheimer’s disease (Miklossy, 2015). Both Aβ and NFT pathologies are currently known to be paired in such neurodegenerative states as post-stroke syndrome (Thiel et al., 2014), Parkinson’s disease (Petrou et al., 2015), traumatic brain injury (Kenney et al., 2018), HIV-dementia (Xu and Ikezu, 2009), Lewy body dementia (Colom-Cadena et al., 2013), and lead poisoning (Li et al., 2010). These disorders appear to be on the same pathophysiological spectrum with Alzheimer’s disease (Morris et al., 2018). Most importantly, considerable Aβ and Tau depositions are present in the individuals with normal cognition (Morris et al., 2018).

For example, in 1997 the unusual case of Sister Mary was reported (Snowdon, 1997) as a representative for the successful aging. Despite her high cognitive capacity at the advanced age of 101 years-old, the autopsy revealed her brain contained numerous senile plaques and NFT, which satisfied the Khachaturian criteria for Alzheimer’s disease (Khachaturian, 1985). Her case is by no means exceptional. One should note that in non-demented aging, up to 40% of the cognitively normal individuals may reach some level of neuropathological criteria for Alzheimer’s disease (Price et al., 2009). Even if the restrictive diagnostic criteria for Aβ and Tau pathology are applied, approximately 20% of the cognitively normal elderly exhibit neuropathological evidence of Alzheimer’s disease (Price et al., 2009). More recently, PET scans show that 10∼30% of the cognitively normal individuals is Aβ-positive, while 18% of older adults is Tau-positive (Chételat, 2013; Chételat et al., 2013; Scholl et al., 2016).

The present clinical and neuropathological diagnostic guidelines are derived from the assumptions that (1) depositions of the amyloids and tangles are more severe in the Alzheimer brain, compared to the brain of subjects with normal cognitive aging, and so, (2) Aβ and Tau depositions should be a specific marker of both preclinical and symptomatic stages of Alzheimer’s disease. Even if an individual presents with the advanced dementia, hippocampal and parietal (especially, precuneus) atrophy on the MRI, and decreased glucose uptake and/or regional blood flow in the parietal lobe on the PET without neuropathological evidence of Aβ pathology, the current view posits that the patient simply does not have Alzheimer’s disease. This is a “consensus” view broadly shared by both the NIA-AA diagnostic guidelines (2011–2018) and the International Work Group (IWG) criteria (2007–2014) (Morris et al., 2018). Although “Aβ pathology” had essentially become synonymous with “Alzheimer’s disease” since the emergence of diagnostic criteria of Alzheimer’s disease in 1980s, the amyloid hypothesis still remains unproven and Aβ is not causally linked to Alzheimer dementia. Now, one had better abandon such a concept that Alzheimer’s disease cannot be pathologically explained without accounting for Aβ and Tau.

Most importantly, presence of amyloid plaques, even diffuse although rare, in cognitively normal individuals (Vinters, 2015) should be explained in detail. One had better keep in mind that Aβ pathology is one of the risk factors for Alzheimer’s disease, because it might be useful for predicting conversion from non-symptomatic or mild cognitive impairment (MCI) to symptomatic stages. However, the presence of Aβ does not always guarantee it, and Aβ pathology alone is incapable of predicting cognitive decline as a disease-defining biomarker. There is another discrepancy between the neuropathology and the clinical phenotype, i.e., some individuals diagnosed with Alzheimer’s disease do not have Aβ pathology, whereas those not meeting pathological criteria were clinically diagnosed with Alzheimer’s disease (Beach et al., 2012). The recent cohort study showed a 30% discrepancy between clinical and biomarker data using a novel blood-based Aβ assay (Nakamura et al., 2018). Some individuals diagnosed with Alzheimer’s disease exhibit non-Aβ pathologies. For example, Aβ pathology-negative patients with a clinical diagnosis of Alzheimer’s disease showed non-Aβ pathologies at autopsy. These included tangle-only dementia, fronto-temporal lobar degeneration, Lewy body dementia, hippocampal sclerosis, etc. (Beach et al., 2012). That means, the diagnosis of Alzheimer’s disease is still imprecise.

Then, what pathological markers show more correlation beyond Aβ and Tau in Alzheimer’s disease?

In the following chapter, we will focus on hydroxynonenal and discuss *ALDH2 gene* transgenic and knock-out mice as precise models of Alzheimer’s disease.

## *ALDH2* gene transgenic and knock-out mice

The research of sporadic (late-onset) Alzheimer’s disease has been hampered by a paucity of precise animal models. Actually, there have been very few rodent models that completely reproduce the complexity of human Alzheimer’s disease. Since *APP* and *APP/PS_1_* transgenic mice models do not show frank neuronal death, their importance in understanding the pathogenesis of Alzheimer’s disease is now questionable by taking into account their poor translational value (Ricciarelli and Fedele, 2017). We suggest that alternative mice models for Alzheimer’s disease that take into account various causes such as genetic, environmental, dietary, and pathological elements should be indispensable.

Oxidative stress leads to generation of many reactive intermediates that overwhelm the antioxidant defense system. When occurring in the brain, oxidative stress was thought to be a causative factor of sporadic Alzheimer’s disease (Butterfield, 1997; Markesbery, 1997; Praticò, 2008; Wang et al., 2014). One of the key enzymes participating in the detoxification not only of ethanol’s metabolite acetaldehyde but also of lipid-peroxidation product hydroxynonenal is the mitochondrial enzyme “aldehyde dehydrogenase 2” (ALDH2: EC 1.2.1.3) (Edenberg, 2007; Oyama et al., 2007; Sun et al., 2014; Joshi et al., 2019). The mitochondrial ALDH2 enzyme catabolizes both ethanol and cooking oil. An inactivating mutation in *ALDH2 gene* causes facial flushing in response to alcohol consumption. However, intake of deep-fried foods which contain abundant hydroxynonenal is not associated with any acute response to cooking oil. As nearly half of East Asians carry Glu504Lys loss of function mutation (ALDH2*2), they are prone to accumulate hydroxynonenal-modified protein adducts in the body. Accordingly, ALDH2*2 was demonstrated to be a risk factor for Alzheimer’s disease especially in East-Asians (Wang et al., 2008; Chen et al., 2019; Joshi et al., 2019). Recent meta-analysis suggested a link between ALDH2*2 and Alzheimer’s disease (Chen et al., 2019). Moreover, a case-control study from Japan indicated that ALDH2*2 was associated with late-onset Alzheimer’s disease (Kamino et al., 2000). The mechanistic insights underlying ALDH2 action were elucidated using murine models with the ablation of the *ALDH2* gene, for example, ALDH2*2 overexpressing mice by Ohsawa et al. (2008), ALDH2*2 knocked-in mice by Zambelli et al. (2014), and *ALDH2^–/–^* mice by D’Souza et al. (2015). These studies showed that deficiency in ALDH2 activities results in elevation of hydroxynonenal with increased cell degeneration/death. Hydroxynonenal levels showed age-dependent increase in the brains of the ALDH2*2 knocked-in mice. Further, hydroxynonenal levels correlated with neurodegeneration and memory loss in these ALDH2*2 overexpressing mice (Ohsawa et al., 2008).

**FIGURE 3:**
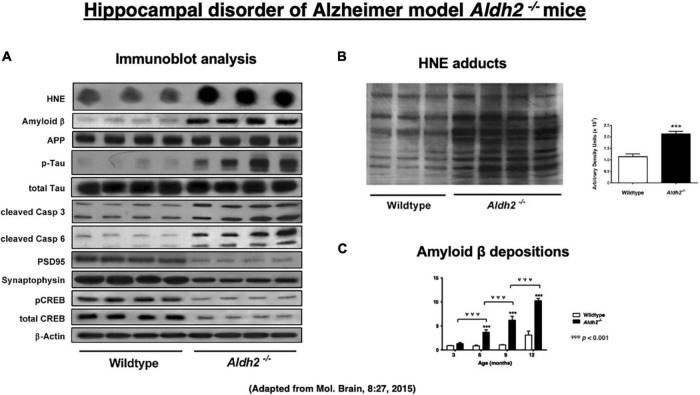
Upregulation of hydroxynonenal (HNE) and Alzheimer’s disease-associated markers **(A)**, HNE adducts **(B)**, and amyloid β **(C)** in the hippocampal homogenates in the *ALDH2^– /–^* mice, compared to the wild-type mice. Adapted from D’Souza et al. (2015).

Since the majority of reactive oxygen species are generated during the mitochondrial metabolism, intake of high-fat diets, bioenergetic activity, and mitochondrial dysfunctions are known to generate excess amounts of oxidant stress (Sun et al., 2014; Yamashima, 2020a). Reactive oxygen species are relatively short-acting, whereas hydroxynonenal is a long-acting oxidative stressor albeit its half-life being less than a few minutes, (Sayre et al., 1997; Ando et al., 1998; Uchida, 2003; Siegel et al., 2007; Butterfield et al., 2010; Bekyarova et al., 2019; Gianazza et al., 2019), because it remains in the body as protein adducts, for example, by binding with atheromatous plaques in the arterial wall and Aβ plaques in the brain. Accordingly, mice lacking *ALDH2* gene (*ALDH2^–/–^*) show increased levels of hydroxynonenal and its adducts in the serum and diverse organs including the brain. D’Souza et al. (2015) characterized *ALDH2^–/–^* mice as a precise model of Alzheimer’s disease, thus suggesting a new insight into the molecular mechanisms driving Alzheimer’s disease. Intriguingly, their *ALDH2^–/–^* mice showed marked increases in hydroxynonenal adducts in the hippocampus (Figure 3B). They showed both age-related increases in Aβ (Figure 3C), p-Tau, and activated caspases, and age-related decreases in p-GSK3β, PSD95, synaptophysin, CREB, and p-CREB (Figure 3A; D’Souza et al., 2015). Most importantly, *ALDH2^–/–^* mice were distinct with the previously reported transgenic model mice, because progressive, age-related memory deficits and cognitive impairments were remarkable early in life at 6.5–7 months but in age-related manner, and showed brain atrophy due to neuronal degeneration and death. Importantly, the latter was rarely observed in the conventional transgenic mice models (Palmer, 2011). The calculated area (mean ± SD) of hippocampus and overlying neocortex showed a significant atrophy (∼15% reduction), being 6.3 ± 0.6 mm^3^ in *ALDH2^–/–^* mice, compared to 7.4 ± 1.0 mm^3^ in the control mice. *ALDH2^–/–^* mice had some characteristics distinct from the mice overexpressing the Glu504Lys mutation of *ALDH2* gene (Ohsawa et al., 2008). Less than 50% of these mice exhibited neurodegeneration in 6 month old, while all *ALDH2^–/–^* mice showed widespread neurodegeneration in 3 month old. Accordingly, memory impairment occurred at 12 months in ALDH2*2 overexpressing mice, whereas it was observed as early as at 3.5–4 months in *ALDH2^–/–^* mice. The latter showed more consistent expression of pathological changes and memory impairment than the ALDH2*2 overexpressing mice (D’Souza et al., 2015).

In summary, both ALDH2*2 overexpressing mice and *ALDH2^–/–^* knock-out mice have advantages over the conventional *APP, PS_1_*, and *PS*_2_ transgenic mice. ALDH2*2 overexpressing mice are precise models of late-onset (sporadic) Alzheimer’s disease, while *ALDH2^–/–^* mice are precise models of early-onset (familiar) Alzheimer’s disease.

## Both ischemic and degenerative neuronal death occurs via hydroxynonenal

What is the root substance of Alzheimer’s disease beyond Aβ and Tau?

Recently, we found that either exogenous or endogenous hydroxynonenal load long-standing oxidative stress not only in the liver and pancreas but also in the brain (Yamashima, 2020a,^2023^; Boontem and Yamashima, 2021; Seike et al., 2022; Yamashima et al., 2023). Hydroxynonenal can accumulate in our body from exogenous sources, e.g., after consumption of deep-fried foods cooked by linoleic acid-rich vegetable oils. Further, reactive oxygen species which oxidize carbon–carbon double bonds (Ayala et al., 2014) of ω-6 PUFA (especially, linoleic and arachidonic acids) at biomembranes generate endogenous hydroxynonenal. Among lipid peroxidation products, hydroxynonenal is the most bioactive lipid alkenals that impede cell health by forming covalent adducts with diverse proteins, nucleic acids, and membrane lipids. Although reactive oxygen species have a relatively short half-life, hydroxynonenal can react with senile or atheromatous plaques and affect lesions far from the initial site for long time, because it is water-soluble, but has also strong lipophilic characteristics, and is slowly metabolized once it forms adducts with macromolecules. Therefore, hydroxynonenal has been considered a mediator of toxic effects as a long-term oxidative stressor (Uchida, 2003; Bekyarova et al., 2019; Gianazza et al., 2019).

For example, hydroxynonenal levels are higher in the hippocampal tissues from the patients with Alzheimer’s disease (Williams et al., 2006), especially within Aβ peptide plaques and NFTs (Sayre et al., 1997; Ando et al., 1998; Siegel et al., 2007; Butterfield et al., 2010). Furthermore, intracellular Aβ_42_ localizes to the inner mitochondrial membrane and leads to generation of excessive reactive oxygen species (Pagani and Eckert, 2011). This enhances lipid peroxidation, thus further increasing the hydroxynonenal concentration (Butterfield et al., 2002). Not only in the brains of patients with Alzheimer’s disease but also in those with Parkinson’s disease, as well as in experimental models relevant to these neurodegenerative disorders, hydroxynonenal accumulates in the brain cells. Hydroxynonenal triggers aggregation of Aβ-peptide and α-synuclein, and causes synaptic dysfunction and neuronal death. In addition, hydroxynonenal-protein adducts are detected in the brains with Lewy body dementia, Down syndrome, and in the spinal cord of the amyotrophic lateral sclerosis (Zhang et al., 2017). Intriguingly, by the consecutive injections of the synthetic hydroxynonenal to the Japanese macaque monkeys, recently Yamashima has succeeded to make diverse brain pathology being characteristic of Alzheimer’s disease such as widespread neuronal degeneration/death, accumulation of numerous autophagolysosomes and multilamellar structures, evidence of lysosomal membrane permeabilization, and loss of the synaptic vesicles, albeit absence of Aβ depositions (Yamashima, 2021). Importantly, most of the ultrastructural characteristics which were observed in the human patients (Nixon et al., 2005) were reproduced in the monkey brain in the circumstances devoid of Aβ.

For the detoxification of hydroxynonenal, three pathways have been described: (1) conjugation with glutathione by glutathione *S*-transferases, (2) reduction by aldo-keto reductases, and (3) oxidation by ALDH2 (Picklo et al., 2001; Oyama et al., 2007; Sun et al., 2014). Hydroxynonenal detoxification by glutathione *S*-transferases declines due to age-dependent enzyme depletion (Schaur et al., 2015); glutathione *S*-transferase activity decreases in the hippocampus of Alzheimer brain (Lovell et al., 1998), and brain levels of superoxide dismutase, catalase, glutathione reductase also decrease in Alzheimer’s disease (Sultana et al., 2013). Aldo-keto reductase is present in the cerebral cortex, hippocampus, basal ganglia, midbrain, and the cerebellum (Picklo et al., 2001). Among the three hydroxynonenal detoxifying pathways, only ALDH2 is increased in the Alzheimer brains (Picklo et al., 2001; Michel et al., 2010). ALDH2 is located in the mitochondrial matrix (Kitagawa et al., 2000), and is most efficient in catalyzing toxic acetaldehyde to carboxylic acid. ALDH2 can also catabolize hydroxynonenal for neuroprotection, because it is widely expressed in the temporal and frontal cortex, hippocampus, and the midbrain (Zimatkin et al., 1992; Stewart et al., 1996; Guo et al., 2013).

**FIGURE 4:**
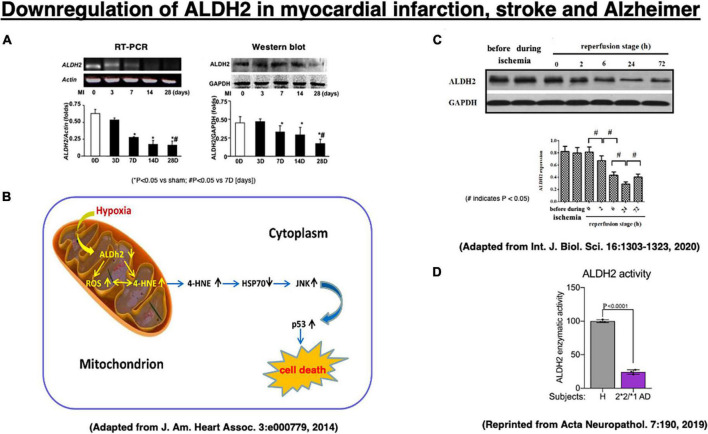
Downregulation of the ALDH2 mRNA and protein in the experimental myocardial infarction **(A,B)** and the stroke model after the middle cerebral artery occlusion/reperfusion **(C)** in rats, and enzymatic activity of ALDH2 in lysates from the ALDH2*2/*1 Alzheimer patient-derived fibroblasts **(D)**. Adapted from Sun et al. (2014), and Xia et al. (2020), and reprinted from Joshi et al. (2019).

Three reports of experimental myocardial infarction and stroke as well as human materials implicate ALDH2 or Hsp70 in cell death (Figure 4). In 2008, we showed direct correlation between ALDH2 activity and infarct size in a model of ischemia and reperfusion using Langendorff isolated heart model, using inhibitors and activators of ALDH2 the *R*^2^ correlation with infarct (Chen et al., 2008). In that study we also found that hydroxynonenal inactivates ALDH2, and a small molecule that we have identified, Alda-1, prevents ALDH2 inactivation. Sun et al. (2014) suggested a role for Hsp70 in hydroxynonenal-induced cardiomyocyte apoptosis. In that model, isolated murine heart was perfused with hydroxynonenal (10 nmol/L) to induce cardiomyocyte injury and death (Sun et al., 2014). Compared to the vehicle treatment, ischemic area was significantly increased on the Nagar-Olsen staining, and TUNEL-positive nuclei were also significantly increased in the hydroxynonenal-treated hearts. Because of lack of apoptotic bodies and presence of membrane disruption, Sun et al. (2014) suggested that hydroxynonenal induces cardiomyocyte cell death through downregulation of ALDH2 (Figure 4A) and activation of c-Jun N-terminal kinase (JNK), and p53, because activation of JNK and p53 was reversed by the transfection of adenovirus-vector encoding Hsp70. They also showed that upregulation of ALDH2 reduced cardiomyocyte injuries after myocardial infarctions via decrease of hydroxynonenal, whereas its downregulation increased the injuries through increase of hydroxynonenal (Figure 4B; Sun et al., 2014).

In addition, Xia et al. (2020) recently demonstrated that downregulation of ALDH2 is related to the occurrence of acute stroke. In the stroke model of middle cerebral artery occlusion/reperfusion in rats, they demonstrated a significant downregulation of ALDH2 expression at 2∼72 h of the reperfusion stage, being lowest at 24 h after reperfusion, compared to the phase of ischemia by Western blotting (Figure 4C). Subsequently, activated JNK was translocated into the nuclei, and this promoted caspase-3 mRNA expression and neuronal apoptosis in the penumbra (Shen and Liu, 2006; Shi et al., 2014). Furthermore, in humans, enzymatic activity of ALDH2 was ∼25% in lysates from ALDH2*2/*1 Alzheimer patient-derived fibroblasts, compared to the control fibroblasts (Figure 4D; Joshi et al., 2019). Both myocardial infarction and acute stroke in rats are the case of acute, intense ischemia of the heart or the brain inducing a similar pattern of ALDH2 downregulation. Although the latter is associated with increments of hydroxynonenal, both of these experimental paradigms did not study the adverse effects of hydroxynonenal itself. We speculate that downregulation of ALDH2 and concomitant increase in hydroxynonenal may occur in the chronic, mild ischemia of the brain at the subclinical stage of Alzheimer’s disease.

In Alzheimer’s disease, occurrence of apoptotic neuronal death is extremely rare; very few hippocampal neurons exceptionally display the morphologic evidence of apoptosis, i.e., dense chromatin condensation called apoptotic bodies (Wyllie et al., 1980). For example, only one among 2,500 to 5,650 hippocampal neurons (0.02–0.05%) showed morphologic evidence of apoptosis and expression of activated caspase-3. Caspase activation does not have a crucial role in the majority of widespread neuronal death occurring in Alzheimer’s disease (Jellinger, 2001). Although caspases were originally identified as important mediators of apoptosis, Yuan et al. (2016) have identified their roles in mediating necrotic cell death. Since we have also failed to observe evidence of apoptosis in the Alzheimer brain, it is highly probable that neuronal death in Alzheimer’s disease occurs not via apoptosis cascade but other cascade of programmed cell death. Concerning the occurrence of ischemic and Alzheimer neuronal death, there should be a common molecular cascade, so this would be discussed in the following chapter by focusing on Hsp70.1 as a lysosomal membrane stabilizer.

## Calpain cleaves carbonylated Hsp70.1 and induces lysosomal membrane disintegration

As described above, we recently found that hydroxynonenal is a common mediator of programmed cell death events in the brain, liver, and pancreas (Yamashima, 2020a, 2023; Boontem and Yamashima, 2021; Seike et al., 2022). These findings lead to two questions: what is the molecular target of carbonylation by hydroxynonenal, and how can it induce cell death in diverse organs? It is likely that a common molecular cascade of cell death being induced by hydroxynonenal is present among three organs (Yamashima et al., 2020).

**FIGURE 5:**
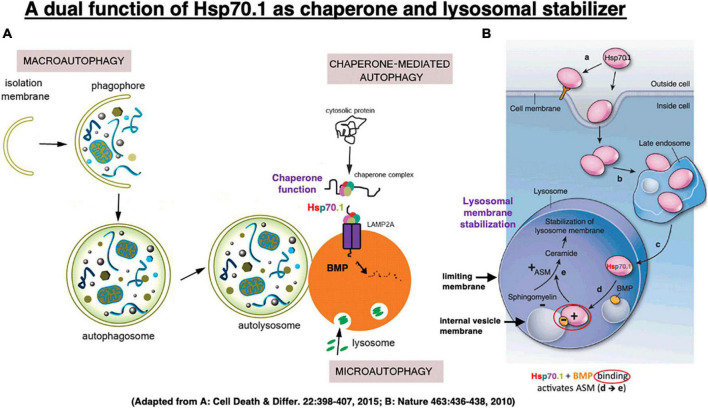
In chaperone-mediated autophagy, Hsp70.1 works as a molecular chaperone which transports garbage proteins and organelles to lysosomal lumen for degradation **(A)**. It also works as a lysosomal stabilizer which facilitates generation of ceramide at internal vesicle membranes via binding with bis(monoacylglycero)phosphate (BMP) and activating acid sphingomyelinase (ASM) **(B)**. Ceramide stabilizes lysosomal membranes by facilitating mutual adhesion of internal vesicles and adhesion of lysosomes with other cytoplasmic components. Adapted from Nikoletopoulou et al. (2015) and Horváth and Vígh (2010).

**FIGURE 6:**
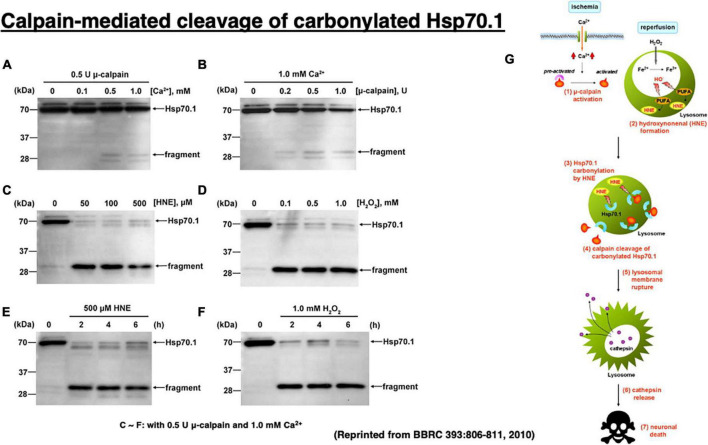
Calpain-mediated cleavage of Hsp70.1 in the hippocampal homogenates of monkeys **(A–F)** and the calpain–cathepsin cascade **(G)** explaining the molecular mechanism of ischemic neuronal death. Since calpain-mediated Hsp70.1 cleavage occurs similarly by extrinsic hydroxynonenal **(C,E)** or H_2_O_2_ **(D,F)**, oxidation of the biomembranes is capable of immediately generating intrinsic hydroxynonenal to carbonylate Hsp70.1. Trigger of the ischemic neuronal death is Ca^2+^ mobilization **(G)**, as it was in Alzheimer neuronal death (Figure 1). In both ischemic and Alzheimer neuronal death, lysosomal cathepsins are executioners when released into the cytosol with protons. Reprinted from Sahara and Yamashima (2010).

The heat-shock protein (Hsp) was discovered by Ritossa (1962) in Italy, studying heat shock in *Drosophila melanogaster*. Hsp is a molecular chaperone which is essential for cells to cope with environmental stresses. Hsp70.1 (also called Hsp70, Hsp72, or HSPA1 in humans) is an evolutionarily highly conserved molecular chaperone that protects stressed cells from various injuries by inhibiting lysosomal membrane disintegrity (Nylandsted et al., 2004; Oikawa et al., 2009; Yamashima and Oikawa, 2009; Kirkegaard et al., 2010; Sahara and Yamashima, 2010; Yamashima, 2023). Hsp70.1 enables neurons to withstand potentially lethal insults through its dual housekeeping function as chaperone protein and lysosomal stabilizer (Figures 5, 6). Hsp70.1 assists protein folding or aggregation, degradation, complex assembly, and translocation to inhibit accumulation of damaged proteins. Damaged/aged proteins destined for degradation enter lysosomes within the basket (substrate-binding domain) of Hsp70.1 via chaperone-mediated autophagy (Figure 5A), and/or macroautophagy and microautophagy (Turk and Turk, 2009). Damaged parts of the cytoplasmic organelles are engulfed within autophagosomes, and ultimately delivered to lysosomes for degradation (Maiuri et al., 2007). Autophagy provides catabolic substrates from the recycling of macromolecules and organelles, and this can facilitate cell survival (Jin and White, 2008).

Hsp70.1 can protect against apoptosis and necrosis by interfering with multiple cell death pathways (Frebel and Wiese, 2006; De Maio, 2014). Although the synthesis of most cellular proteins is downregulated at the onset of brain injury, the levels of functional Hsp70.1 are actually upregulated in certain neurons. For example, in the monkey model of transient global brain ischemia, non-oxidized Hsp70.1 remains in the ischemia-resistant CA3 neurons and the dentate granular cells of the hippocampus, but carbonylated Hsp70.1 increases in ischemia-vulnerable CA1 neurons (Tsuchiya et al., 2003; Oikawa et al., 2009; Yamashima and Oikawa, 2009; Mori et al., 2020). Neuroprotective effects of Hsp70.1 were demonstrated to reduce lesion sizes and result in better neurological outcomes in stroke models overexpressing this protein by viral vectors and in overexpressing transgenic mice (Lee et al., 2001, 2004; Batulan et al., 2006; Kim et al., 2016). A direct delivery of exogenous Hsp70.1 to the brain in a rodent stroke model reduced infarct volumes, improved neurological deficits, and led to survival of neural progenitors, but the target molecules that Hsp70.1 interacted have not been identified (Doeppner et al., 2009; Zhan et al., 2010).

Kirkegaard et al. demonstrated that Hsp70.1 in cancer cells is a “molecular chaperone” that facilitates folding of newly formed polypeptides, and promotes cell survival by inhibiting the breakdown of lysosomes and enhancing lysosomal membrane stability after the translocation of Hsp70.1 to the lysosomal lumen (Figure 5B; Kirkegaard et al., 2010). Specifically, Horváth and Vígh (2010) reported that when recombinant Hsp70 was added to cancer cells (Figure 5B [**a**]), it is transported via late endosome [**b**] into the acidic lysosome [**c**] by endocytosis, where it interacts with an anionic phospholipid, bis(monoacylglycero)phosphate (BMP)[**d**]. BMP phospholipids are predominantly localized to the internal lysosomal membranes and serve as cofactors for the enzyme acid sphingomyelinase (ASM: EC 3.1.4.12). The substrate-binding domain of Hsp70.1 controls its interaction with BMP and hence lysosomal stabilization. Hsp70.1-BMP interaction enhances binding of BMP with ASM, and activates this enzyme to catalyze hydrolysis of sphingomyelin (ceramide phosphorylcholine) into ceramide [**e**] and phosphorylcholine (Smith and Schuchman, 2008). In internal vesicles and membranes of lysosomes, BMP is negatively charged at pH 4.2. As ASM has an isoelectric point of pH 6.8, it has positively charged regions in the acidic environment which enable its binding of negatively charged BMP at the internal vesicle membrane (Kölzer et al., 2004; Schulze et al., 2009).

The ceramide that is generated by ASM-dependent hydrolysis of the membrane-bound sphingomyelin, serves as a backbone for complex sphingolipid-based structural lipid in the membrane macrodomains (Hannun and Obeid, 2008). ASM mediates increase in the lysosomal ceramide, and this facilitates fusion of lysosomes with intracellular vesicles and the plasma membrane (Heinrich et al., 2000; Goñi and Alonso, 2002; Utermöhlen et al., 2008) through modification of the conformation of lysosomal limiting membrane. Conversely, various apoptotic stimuli induce the translocation of ASM to the plasma membrane outer leaflet to activate membrane-associated signaling molecules involved in apoptosis (Smith and Schuchman, 2008). Thus, depending on whether ceramide is generated inside the lysosome or at the plasma membrane, it has opposing effects on cell survival. When cells are engineered to overproduce Hsp70.1, the cells show higher ASM activity than did their normal counterparts, and are protected against stress-induced lysosomal damage. In contrast, inhibition of Hsp70-BMP binding reduces ASM activity causing lysosomal membrane destabilization, and abolishing Hsp70.1’s cytoprotective effect (Kirkegaard et al., 2010; Petersen et al., 2010; Petersen and Kirkegaard, 2010). Binding of Hsp70.1 to BMP is essential for the lysosome-stabilizing effect of Hsp70.1 by regulating sphingomyelin metabolism (Figure 5).

It is almost impossible to identify the *in vivo* substrate of activated μ-calpain in the living animals, because calpain cuts the substrate protein presumably within seconds of contact. Therefore, calpain-mediated Hsp70.1 cleavage was studied *in vitro*, by comparing normal and oxidized tissues of the monkey hippocampal CA1 (Figure 6; Sahara and Yamashima, 2010). In that system, Hsp70.1 being involved in the oxidized CA1 tissue becomes vulnerable to the μ-calpain-mediated cleavage than in the normal tissue and this effect was a little bit increased in the presence of increasing amounts of CaCl_2_ (Figures 6A, B). In contrast, in the presence of either hydroxynonenal (HNE) or H_2_O_2_, Hsp70.1 was cleaved to generate much more 30 kDa Hsp70.1 fragments (Figures 6C, D) regardless of the incubation time (Figures 6E, F). In the presence of such oxidative stressor, carbonylated Hsp70.1 was efficiently cleaved by activated μ-calpain. Not only CA1 but also other monkey brain tissues such as thalamus, putamen, and medulla oblongata showed the same results (Liang et al., 2016).

**FIGURE 7:**
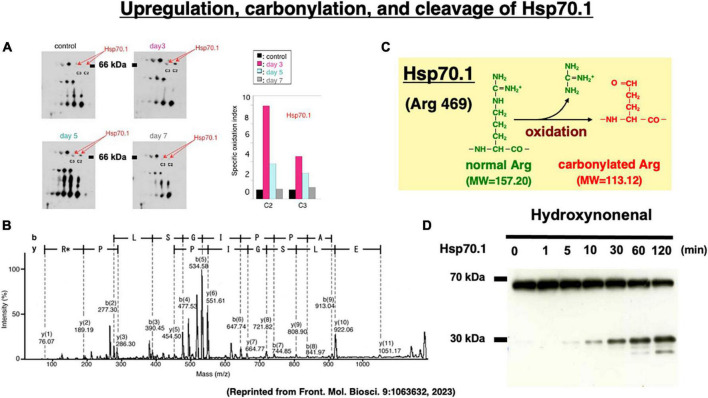
Upregulation of carbonylated Hsp70.1 on the postischemic day 3 and day 5 **(A)**, and carbonylation at the key site Arg469 (R*) of Hsp70.1 **(B,C)** on the proteomics analysis, in the monkey hippocampal CA1 tissues after transient global brain ischemia. Time-dependent increase of calpain-mediated Hsp70.1 cleavage by the *in vitro* hydroxynonenal treatment **(D)**. Reprinted from Yamashima (2023).

Two-dimensional gel electrophoresis for the immunoblot detection of carbonylated protein analysis (2D Oxyblot) after immunoprecipitation with anti-Hsp70.1 antibody, showed a marked upregulation of carbonylated Hsp70.1 on the postischemic days 3 (pink) and 5 (blue) of the hippocampal CA1 tissue after transient ischemia, compared to the control (black) (Figure 7A). The specific oxidation index was significantly increased on the postischemic days 3 and 5 (Figure 7A). Matrix-assisted laser desorption ionization-time of flight/time of flight (MALDI-TOF/TOF) analysis with the Mascot search identified the carbonylated peptide ion (459-FELSGIPPAPR*G-470) and the y2 fragment ion atm/z 113.12, as carbonylation of Arg469 in Hsp70.1 (Figure 7B). The decrease of its molecular weight from 157.20 to 113.12 indicates carbonylation, i.e., removal of three amino groups from Arg469 (Figure 7C). Arg469 of Hsp70.1 is the key site responsible for hydrogen bonding between the substrate-binding β-sheet domain and its lid, α-helix domain (Zhang et al., 2014). In addition, using various normal brain tissues, the calpain-mediated cleavage of the carbonylated Hsp70.1 was demonstrated to occur *in vitro* simultaneously with hydroxynonenal-induced carbonylation (Figure 7D; Yamashima et al., 2014; Liang et al., 2016). These data altogether indicated that Hsp70.1 is an *in vivo* substrate of activated μ-calpain in the living brain especially after the oxidative stress.

## “*Calpain–cathepsin hypothesis*” can explain both ischemic and Alzheimer neuronal death

During the past three decades, the “lysosomal membrane permeabilization/rupture” has emerged as a prominent area of research to elucidate the mechanisms of programmed cell necrosis (Yamashima, 2023). The concept of “lysosomal membrane permeabilization (LMP)” was first described *in vitro* in the oxidative stress-induced apoptosis of cultured glioma cells by Öllinger and Brunk (1995), Brunk et al. (1997), Roberg and Öllinger (1998), and Brunk and Svensson (1999). In contrast, using monkeys subjected to transient global brain ischemia, Yamashima et al. for the first time observed “lysosomal membrane rupture” *in vivo* in the delayed necrosis of the hippocampal CA1 neurons (Yamashima et al., 1996, 1998, 2023; Yamashima, 2000, 2023; Oikawa et al., 2009; Yamashima and Oikawa, 2009; Sahara and Yamashima, 2010).

**FIGURE 8:**
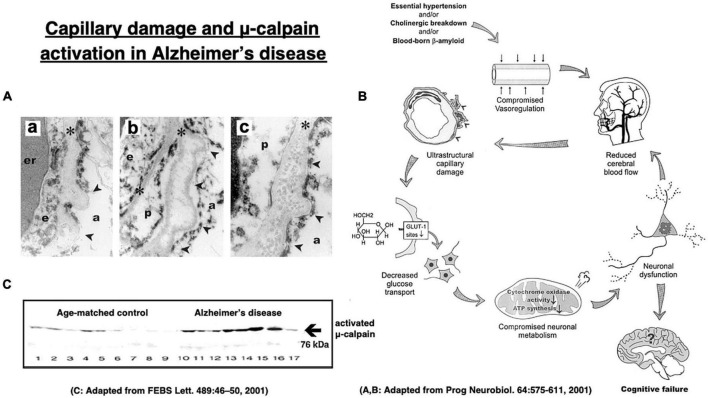
Age-dependent capillary wall thickening in Alzheimer’s disease **(A)** with the resultant impairment of brain microcirculation **(B)**, and μ-calpain activation **(C)** in the brain with Alzheimer patient. **(a)** shows basement membrane (asterisks) thickening, **(b)** shows its splitting, and **(c)** shows fibril depositions in the basement membrane in the capillary wall **(A)**. er, erythrocyte; e, endothelial cells; p, pericyte; arrowheads, sites of basement membrane pathology; a, astrocytic endfoot. These altogether induces impairments of microcirculation and glucose transport **(B)**. Long-standing brain ischemia and/or hypoglycemia induce more intense μ-calpain activation in the patients with Alzheimer’s disease (cases 10∼17), relative to the age-matched, healthy individuals (cases 1∼9). However, calpain activation alone cannot induce Hsp70.1 cleavage, and Hsp70.1 oxidation is indispensable for the activated μ-calpain to induce its cleavage (Figures 6A–F). Adopted from Farkas and Luiten (2001) and Taniguchi et al. (2001).

More than 70% of Alzheimer patients show cerebral amyloid angiopathy which narrows the small vessels and causes cerebral ischemia (Figure 8A; Farkas and Luiten, 2001; Cullen et al., 2006; Hardy and Cullen, 2006; Smith and Greenberg, 2009). The cerebral blood flow of Alzheimer patients was reported to be approximately 80–90%, compared to the age-matched controls (Farkas and Luiten, 2001). Intriguingly, in 1907 Dr. Alois Alzheimer described in his first case evidence of microvascular changes such as “endothelial proliferation” and “neovascularization.” Later, Fischer et al. (1990) confirmed a striking and statistically significant reduction in the vascular net density specifically in the Alzheimer brains. Both Aβ- and oxidative stress- induced inflammatory damages to capillaries were thought to be critical for the impairments of cerebral microcirculation and glucose transport due to the basement membrane thickening in the patients with Alzheimer’s disease (Figure 8B; Farkas and Luiten, 2001; Marchesi, 2011). The Alzheimer brains exhibit vascular pathology such as cerebral infarcts, microinfarction, white matter changes, and even hemorrhages (Kalaria, 1996, 2000; Kalaria and Ballard, 1999). Therefore, due to the long-standing ischemia (hypoglycemia) of the brain, μ-calpain activation occurs in the Alzheimer brain approximately seven-fold more intense, compared to the age-matched, non-demented control subjects (Figure 8C; Taniguchi et al., 2001).

*In vitro* cleavage of Hsp70.1 in brain tissues by activated μ-calpain suggests that Hsp70.1 modulates calpain-mediated lysosomal membrane rupture/permeabilization after ischemia/reperfusion [Figure 6G (**1**)–(**7**)]. During the ischemic phase, extensive Ca^2+^ mobilization occurs specifically in the ischemia-vulnerable neuron like CA1, and μ-calpain is excessively activated (**1**). However, calpain activation alone cannot cleave Hsp70.1 without oxidative modification of Hsp70.1 (Figures 6A–D). During the reperfusion phase, oxidative stress induces surplus H_2_O_2_ which is converted to hydroxyradicals by the Fenton reaction within lysosomes. Oxidation of ω-6 PUFA by hydroxyradicals generates endogenous hydroxynonenal (HNE) (**2**) that carbonylates Hsp70.1 at the lysosomal membrane (**3**). Carbonylated Hsp70.1 is then cleaved by activated μ-calpain (**4**) which leads to the lysosomal membrane disintegrity (**5**). Consequently, release of hydrolytic enzyme cathepsins from lysosomes occurs (**6**) to induce delayed neuronal death (**7**).

Cathepsins are lysosomal cysteine proteases which regulate cell proliferation, invasion, and apoptosis, while they have been implicated in cancer, tumor angiogenesis, and neurodegeneration (Stahl et al., 2007). By the immunohistochemistry and the enzyme assay, neuronal lysosomes were demonstrated to contain cathepsins B and L. When released from the lysosome into the cytoplasm, these cathepsins may damage cellular constitutive proteins and cytoskeletons. Simultaneously, they damage the lysosomal membrane from outside, or activate phospholipases that degrade all types of cellular membranes. In addition, they attack mitochondria to generate more H_2_O_2_ by interfering with the mitochondrial electron-transporting complexes (Yamashima et al., 1996, 1998; Yamashima, 2013, 2016).

The oxidative stress hypothesis of Alzheimer’s disease (Butterfield, 1997; Markesbery, 1997; Praticò, 2008) suggested that oxidative damage may play a crucial role for the occurrence of this disease. Our studies above, identifying the molecular events following oxidative stress, led us to formulate the “*calpain–cathepsin hypothesis*” (Yamashima et al., 1998; Yamashima, 2000; Oikawa et al., 2009; Yamashima and Oikawa, 2009). Although the “*calpain–cathepsin hypothesis*” was originally formulated for ischemic neuronal death, we thought it can be applied also for diverse degenerative neuronal death. For example, in the brain with Alzheimer’s disease, increased μ-calpain activation and extralysosomal cathepsin translocation are well known (Cataldo and Nixon, 1990; Saito et al., 1993; Nakanishi, 2003). Further, activation of the calpain–cathepsin cascade was recently confirmed in the patient brain with sporadic Creutzfeldt-Jakob disease (Llorens et al., 2017). In addition, as a mechanism of the *N*-methyl-*N*-nitrosourea-induced photoreceptor cell death in mice, this cascade was demonstrated (Koriyama et al., 2014).

The hypothesis that lysosomal leakage of cathepsins B and L to the cytosol may lead to novel targets for drug discovery to treat neurodegenerative diseases. Until now, many selective cathepsin inhibitors such as CA-074 and E-64c,d with limited adverse effects were developed and showed neuroprotection in the cerebral ischemia models of monkeys (Yamashima et al., 1998) and are awaiting validation in the clinical setting for Alzheimer’s disease (Hook et al., 2020). Since both μ-calpain and cathepsins B, L have a critical function in the physiological conditions, inhibition of the excessive activation with preserving optimal functions is preferable, but difficult especially in the human patients. We speculate that Ca^2+^ channel blocking, combined with hydroxynonenal detoxification by ALDH2 activation, may be a more relevant therapeutic strategy for Alzheimer’s disease, compared to the cathepsin inhibition (Anekonda and Quinn, 2011; Popugaeva and Bezprozvanny, 2013; Yamashima, 2020b).

## The role of exogenous hydroxynonenal in health and disease

Since hydroxynonenal accumulates in the body from the exogenous source, e.g., after eating deep-fried foods and/or high-fat diets, these data suggest that avoiding such food may be salutary. Hydroxynonenal is generated during deep-frying or burn of the ω-6 PUFA-rich vegetable oils. Accordingly, intake of the excessive deep-fried/heated foods or high-fat diets may lead to an elevation of the hydroxynonenal concentration in both the serum and organ (Thaler et al., 2012; Yamashima, 2020b). Both hydroxynonenal and its protein adducts were reported to accumulate in the brains of patients with Alzheimer’s disease (Lovell et al., 1997; Montine et al., 1997; Sayre et al., 1997; Markesbery and Lovell, 1998; McGrath et al., 2001; Fukuda et al., 2009; Reed et al., 2009; Butterfield et al., 2010). Hydroxynonenal is an appropriate indicator of lipid peroxidation in Alzheimer’s disease (Figure 2C), and shows a positive correlation with Aβ_42_ (Figure 2D; Rani et al., 2017).

**FIGURE 9:**
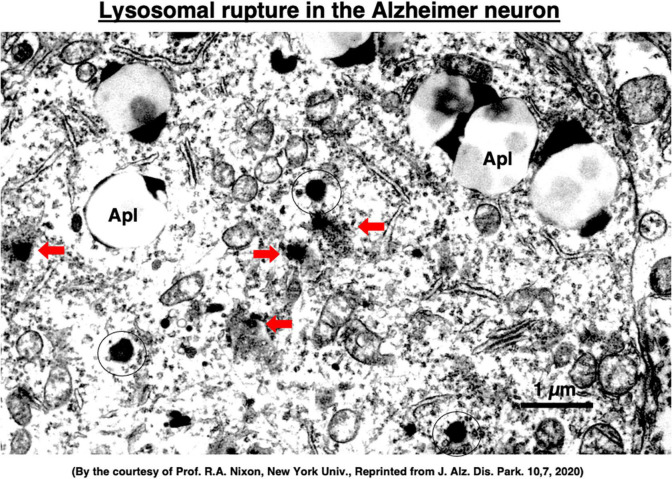
Ultrastructural evidence of the lysosomal membrane permeabilization/rupture (red arrows) compared to the normal lysosomes (circles), and accumulation of autophagolysosomes (Apl) in the cortical neuron of a human patient with Alzheimer’s disease. A number of giant autophagolysosomes (Apl) indicates an incomplete processing of abundant garbage of both protein and lipid degradation products in the lysosomes of the degenerating neuron. In the degenerating neuron of Alzheimer’s disease, mitochondria often show partial disruption of the outer membrane, while endoplasmic reticula are thin and depleted. Reprinted from Yamashima (2020a).

**FIGURE 10:**
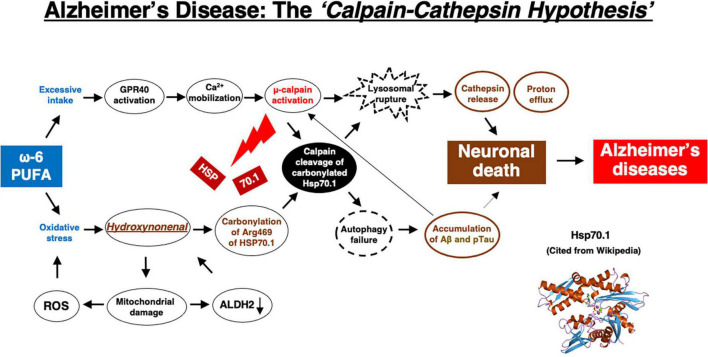
The flow chart of “*calpain–cathepsin hypothesis*” [formulated first by Yamashima et al. (1998), and modified by Yamashima and Oikawa (2009)], showing that ω-6 PUFA can be a trigger of neuronal death in Alzheimer’s disease. ω-6 PUFA contributes to both activation of μ-calpain via GPR40 and generates hydroxynonenal via deep-frying. Hydroxynonenal is a real culprit of neuronal death, whereas amyloid β (Aβ) and phosphorylated Tau (pTau) proteins are byproducts, being generated from autophagy failure due to Hsp70.1 disorder. Aβ indirectly contributes to neuronal death by facilitating μ-calpain activation. Regardless the presence of Aβ or pTau, μ-calpain activation is prone to occur in the aged individuals by the long-term cerebral ischemia due to arteriosclerosis. In the younger people μ-calpain activation may occur via GPR40 overactivation by the intake of excess fatty acids and/or deep-fried foods. Simultaneously, Hsp70.1 is carbonylated by exogenous and/or endogenous hydroxynonenal. So, not only in aged but also in young people, calpain-mediated cleavage of carbonylated Hsp70.1 may cause neuronal death by the lysosomal membrane permeabilization/rupture with the resultant extra-lysosomal leakage of cathepsins and protons. The serum concentration of hydroxynonenal is increased especially in the older people who are associated with the age-dependent ALDH2 deficiency, because they have been affected by the circumferential oxidative stress for long years. Further, hydroxynonenal, especially its adducts, may bind with amyloid β in the senile plaques of the affected brain, and work as a long-term oxidative-stressor. Then, both calpain activation and Hsp70.1 carbonylation occur more drastically within the brain of older people.

Although extremely rare to encounter in the advanced Alzheimer’s disease, Yamashima, with the aid of Prof. R. A. Nixon in New York, found evidence of LMP in the electron-microphotographs of the cortical neurons of the Alzheimer patient (Figure 9; Nixon et al., 2005; Yamashima, 2013, 2016, 2020a, 2021, 2023). In addition, by injecting the synthetic hydroxynonenal to monkeys, Yamashima (2021) could make similar ultrastructural disorders of lysosomes and widespread neuronal death as seen in Alzheimer’s disease. He suggested that free fatty acid receptor, GPR40 in the brain is related to extensive Ca^2+^ mobilization in response to excessive fatty acids. Simultaneously, circumferential oxidative substance may cause oxidation of ω-6 fatty acids with the resultant generation of endogenous hydroxynonenal at biomembranes. As Hsp70.1 is a stress-induced protein and also a lysosomal stabilizer that confers cell protection against stimuli, our data indicate that calpain-mediated cleavage of carbonylated Hsp70.1 causes degenerative neuronal death via the lysosomal rupture and the concomitant autophagy failure (Figure 10). In turn, lysosomal rupture induces both release of cathepsin enzymes which causes breakdown of the cell constitutive proteins, and efflux of the protons which causes decrease of the cytosolic pH thus enabling cathepsin-mediated uncontrolled proteolysis of the cell.

Hydroxynonenal is likely a root substance of Alzheimer’s disease, and Aβ and Tau accumulation may be merely byproducts of lysosomal and autophagy failure which were caused by the calpain-mediated cleavage of the oxidized Hsp70.1 (Figure 10). At present, however, it is difficult to determine whether the source of hydroxynonenal production is exogenous (e.g., incorporated into the serum via deep-fried foods and/or high-fat diets) or endogenous (e.g., generated at biomembranes by the circumferential and/or intrinsic oxidative stress). Regardless, in addition to avoiding consumption of linoleic acid-rich cooking oils to reduce the aldehydic load in the brain, compounds that correct ALDH2 deficiency and activate ALDH2, such as Alda-1 (Chen et al., 2008), or supplementary acetic acid bacteria or yeast containing pure ALDH2 enzymes (Yamashima, 2020b), may provide a therapeutic strategy to slow down or reduce Alzheimer’s disease burden in the world’s aging population. We suggest that a paradigm shift in our assumptions related to the molecular mechanism leading to the neuropathology of Alzheimer’s disease will likely lead to development of better therapeutic intervention.

Lastly, we would like to reconfirm a few issues with hydroxynonenal due to its high reactivity, instability, short half-life, and interaction with various cell macromolecules. In addition, there are multiple underlying causes and factors (Gadhave et al., 2020) such as chronic inflammation, genetic variant, insulin resistance, circumferential factors (air pollution and electromagnetic waves), or lifestyle (lack of exercise, alcohol and drug abuse, and smoking) that contribute to the disease pathogenesis. Therefore, we should keep in mind that avoiding ω-6 PUFA-rich cooking oils may be salutary for preventing Alzheimer’s disease, but that the therapeutic efficacy of hydroxynonenal inhibitors in the future clinical trials would be multifactorial. Targeting hydroxynonenal alone may not be sufficient to achieve substantial benefits at the stage of advanced neuronal death.

## Summary

“*Our major goal must be the prevention of Alzheimer’s disease, and achievement of this goal requires that we first understand its cause*,” described by Katzman (1986).

This is true even now, nearly four decades later. Despite considerable progress in the research about the programmed cell death, surprisingly the intracellular cascade leading to neuronal demise in Alzheimer’s disease still remains incompletely elucidated. The nature, time course, and molecular causes of neuronal death in Alzheimer’s disease are still unresolved by focusing merely on Aβ. Nevertheless, there is increasing evidence for implications of lipid-peroxidation products in the occurrence of Alzheimer neuronal death. We speculate that neuronal death in Alzheimer’s disease is essentially not degenerative but ischemic in nature, being caused synergically by the excessive intake of ω-6 PUFA-rich vegetable oils for oxidizing Hsp70.1 and long-standing brain ischemia due to age-dependent arteriosclerosis or Aβ deposition in the capillary wall to facilitate μ-calpain activation. Roles of hydroxynonenal, Hsp70.1, μ-calpain and cathepsins are indispensable for the occurrence of neuronal death in Alzheimer’s disease. By focusing on these molecular players, the precise mechanism whereby lysosomal membrane disintegrity is induced for neuronal death would be grossly uncovered. Hopefully, the search for hydroxynonenal modulators would contribute to the discovery of novel therapeutic drugs or preventative supplementary compounds. By making a paradigm shift from Aβ to hydroxynonenal, one had better reconsider Alzheimer’s disease. It is still not too late.

## Author contributions

TY: writing, editing, and generation of Figures 1–10. TY, TS, and EM: monkey experiments. DM-R, C-HC, and MK: checking and editing the English language. All authors contributed to the article and approved the submitted version.
